# Strong Humoral but Not Cellular Immune Responses against SARS-CoV-2 in Individuals with Oncohematological Disease Who Were Treated with Rituximab before Receiving a Vaccine Booster

**DOI:** 10.3390/cancers14225537

**Published:** 2022-11-10

**Authors:** Montserrat Torres, Magdalena Corona, Sara Rodríguez-Mora, Guiomar Casado-Fernández, Alejandro Zurdo-Castronuño, Elena Mateos, Fernando Ramos-Martín, Clara Sánchez-Menéndez, María Aranzazú Murciano-Antón, Javier García-Pérez, José Alcamí, Mayte Pérez-Olmeda, Mayte Coiras, Javier López-Jiménez, Valentín García-Gutiérrez, on behalf of the Multidisciplinary Group of Study of COVID-19 (MGS-COVID)

**Affiliations:** 1 Immunopathology Unit, National Center of Microbiology, Instituto de Salud Carlos III, 28220 Majadahonda, Spain; 2 Hematology and Hemotherapy Service, Instituto Ramón y Cajal de Investigación Sanitaria (IRYCIS), Hospital Universitario Ramón y Cajal, 28034 Madrid, Spain; 3 Faculty of Sciences, Universidad de Alcalá, 28871 Alcalá de Henares, Spain; 4 Biomedical Research Center Network in Infectious Diseases (CIBERINFEC), Instituto de Salud Carlos III, 28220 Majadahonda, Spain; 5 Family Medicine, Centro de Salud Doctor Pedro Laín Entralgo, 28924 Alcorcón, Spain; 6 AIDS Immunopathology Unit, National Center of Microbiology, Instituto de Salud Carlos III, 28222 Majadahonda, Spain; 7 Serology Service, Instituto de Salud Carlos III, 28029 Madrid, Spain

**Keywords:** COVID-19 vaccine, anti-CD20, rituximab, hematological malignancies, cytotoxic response, humoral response

## Abstract

**Simple Summary:**

Anti-CD20 treatments produce a prolonged B-cell aplasia that is responsible for a suboptimal humoral response after vaccination against SARS-CoV-2, even months after receiving therapy. However, there is scarce information on the cellular immune response. In this study, we analyzed both cellular and humoral immune responses against SARS-CoV-2 in 18 patients treated with rituximab after receiving a COVID-19 vaccine booster dose. These studies are essential to design an efficient vaccination schedule for these individuals. Although we did not observe a significant benefit in the cellular immune response, there was an increase in the humoral response after the booster dose in rituximab-treated patients in comparison with the response after the second dose, likely due to a longer period of time since the last treatment with rituximab (9.6 versus 13.8 months, respectively). This excellent humoral response was observed even with reduced levels of total B cells, and it was likely responsible for the prevention of severe disease in individuals who acquired a breakthrough infection.

**Abstract:**

The humoral immune response developed after receiving the full vaccination schedule against COVID-19 is impaired in individuals who received anti-CD20 therapy 6–9 months before vaccination. However, there is little information about the cellular immune responses elicited in these individuals. In this study, we analyzed the humoral and cellular immune responses in 18 individuals with hematological disease who received the last dose of rituximab 13.8 months (IQR 9.4–19) before the booster dose. One month after receiving the booster dose, the seroconversion rate in the rituximab-treated cohort increased from 83.3% to 88.9% and titers of specific IgGs against SARS-CoV-2 increased 1.53-fold (*p* = 0.0098), while the levels of neutralizing antibodies increased 3.03-fold (*p* = 0.0381). However, the cytotoxic activity of peripheral blood mononuclear cells (PBMCs) from rituximab-treated individuals remained unchanged, and both antibody-dependent cellular cytotoxicity (ADCC) and direct cellular cytotoxicity (CDD) were reduced 1.7-fold (*p* = 0.0047) and 2.0-fold (*p* = 0.0086), respectively, in comparison with healthy donors. Breakthrough infections rate was higher in our cohort of rituximab-treated individuals (33.33%), although most of the infected patients (83.4%) developed a mild form of COVID-19. In conclusion, our findings confirm a benefit in the humoral, but not in the cellular, immune response in rituximab-treated individuals after receiving a booster dose of an mRNA-based vaccine against COVID-19.

## 1. Introduction

B-cell-depleting therapies have been associated with impaired vaccine responses [1,2,3]. Therefore, patients receiving anti-CD20 treatments, such as rituximab, are at increased risk of infection, even after being vaccinated [4,5]. Rituximab is an anti-CD20 monoclonal antibody that was approved by the FDA in 1997 and is currently used for the treatment of oncohematological diseases such as CD20 positive B-cell non-Hodgkin’s lymphoma (NHL) and chronic lymphocytic leukemia (CLL), as well as inflammatory autoimmune disorders such as rheumatoid arthritis and pemphigus vulgaris [6]. CD20 is a non-glycosylated phosphoprotein that is expressed on the surface of all mature B cells [7], both normal and malignant, which may trigger complement-mediated cytotoxicity, antibody-dependent cell-mediated cytotoxicity (ADCC), and apoptosis in these cells [8]. Therefore, it has been estimated that 48% of individuals with NHL who received rituximab monotherapy experienced grade 3 and 4 cytopenias that could last for 2 weeks, with lymphopenia being the most common. This percentage may be increased when rituximab is combined with other myelosuppressive regimens such as cyclophosphamide, doxorubicin, vincristine, and prednisone (CHOP) and rituximab with CHOP (R-CHOP) [9]. Due to this B-cell-depleting activity, individuals treated with rituximab present impaired immune reactions to different vaccines, such as those against influenza and pneumococcus [10]. In addition, these individuals are at higher risk of progressing to severe outcomes from the Coronavirus Disease 2019 (COVID-19) caused by the emergent coronavirus SARS-CoV-2 in the pre-vaccination era [11]. Therefore, the vaccination of individuals receiving immunosuppressive or anticancer agents has been considered a priority since the approval of COVID-19 vaccines by the National Health Regulatory Authorities [12]. The first two mRNA-based vaccines approved by the US Food and Drug Administration (FDA) in December 2020 by Emergency Use Authorization (EUA) were COMIRNATY (Pfizer-BioNTech) and Spikevax (Moderna) [13,14]. Since then, the administration of these and other authorized vaccines against COVID-19 have led to a huge benefit for the population by arresting the rise in the global number of SARS-CoV-2 cases. Pfizer and Moderna vaccines share many commonalities as they have analogous mechanisms of action [15], they require to be administered in two separate doses to develop an efficient immune response [16], and the efficacy reported for COMIRNATY (95%) [17] is similar to that reported for Spikevax (94.5%) [18]. Although not all real-life studies consider both humoral and cellular immune responses to evaluate the efficacy of COVID-19 vaccines, the presence of neutralizing antibodies after vaccination has been associated with protection against infection [19]. The vaccine efficacy against severe COVID-19 remains high (≥80%) for up to 6 months after vaccination; however, from this point, a progressive decline has been observed [20,21] that is even lower in immunocompromised individuals in comparison with healthy controls (79–90.2% vs. 84–100%, respectively) [22]. Therefore, a booster or third dose of COVID-19 vaccine was approved to induce additional protection against SARS-CoV-2 infection [23] without causing an increased risk of adverse events or systemic reactions [24]. Consequently, the use of both mRNA-based vaccines for booster vaccinations in persons aged 18 years and over and to people with severely weakened immune systems was authorized for COMIRNATY at a full dose and for Spikevax at half the dose, at least 28 days after their second dose [25].

Although vaccines against COVID-19 have proven to be quite effective to avoid severe SARS-CoV-2 illness among immunocompetent individuals, there is still scarce information about the immune response elicited in immunocompromised patients because they were excluded from clinical trials [17,26,27]. Recent real-life studies have established an overall seroconversion rate of 40% after vaccination with two doses against COVID-19 in individuals who received rituximab, but with a wide range that varies from 0% to 80%, depending on the time interval since the last dose of rituximab, type of disease, or B-cell-depletion therapy [28]. In fact, administration of rituximab between vaccine doses impairs the correct development of a functional immune response ([29]), and an interval of at least 9 months between the last rituximab infusion and the vaccination has been recommended to achieve better results [30]. Most studies have focused exclusively on the humoral response, with scarce evidence integrating both cellular and humoral responses, which is known to be essential to control COVID-19 infection and avoiding progression to more severe disease [31,32]. In the absence of an effective humoral response, it is essential to evaluate the efficacy of the cellular response due to it potentially protecting from the fatal outcomes of COVID-19 [33], as well as contributing to a rapid resolution of the infection by eliminating the infected cells [34]. It has been described that T-cell responses may be effective after receiving a booster dose of COVID-19 vaccine in individuals treated with rituximab 9 months ago, even in the absence of a detectable humoral response, but these results were limited to the measurement of cytokines release after peptide stimulation [30].

In this study, we performed a more exhaustive characterization of both cellular and humoral immune responses developed by individuals with hematological diseases who were treated with rituximab and then were vaccinated against COVID-19 with the complete schedule and the booster vaccine dose, measuring the ability to produce an effective cytotoxic, antiviral response.

## 2. Materials and Methods

### 2.1. Study Population

Eighteen individuals with hematological diseases were recruited for this study at the Hematology and Hemotherapy Service of the University Hospital Ramón y Cajal in Madrid (Spain) between September and October 2021. Sixteen individuals were diagnosed with NHL, one individual was diagnosed with immune thrombocytopenia (ITP) associated with systemic lupus erythematosus (SLE), and one individual was diagnosed with autoimmune hemolytic anaemia (AIHA) associated with a myelodysplastic syndrome (DMS). All these individuals received rituximab as part of a therapeutic regimen received for the treatment of their haematological disease. The inclusion criteria were to be over 18 years old, to not have been previously infected with SARS-CoV-2, which was determined through clinical history and a personal interview, and to have been vaccinated through the Spanish Vaccination Program with two doses and a booster dose of the mRNA vaccine Spikevax (mRNA-1273, Moderna). Fifteen healthy donors matched in age and gender and with the same inclusion criteria who had been vaccinated with two doses and a booster dose of the mRNA vaccine COMIRNATY (mRNA BNT162b2, BioNTech–Pfizer) were recruited at the Primary Healthcare Center Doctor Pedro Laín Entralgo in Alcorcón (Madrid, Spain). It has been described that the efficacy of COMIRNATY [17] is similar to that reported for Spikevax [18], which enabled comparing the results obtained in both cohorts in terms of efficacy of the elicited immune response.

Two peripheral blood samples were collected for this study; the first sample was collected the same day all individuals received the booster dose, which was administered 4 months after receiving the second dose, while the second sample was collected 1 month after receiving the booster dose.

### 2.2. Ethical Statement

This study was approved by the Ethics Committee of the Hospital Universitario Ramón y Cajal (favorable Report with reference #053-21), and all individuals gave informed written consent to participate in accordance with the Helsinki declaration. Confidentiality and anonymity were protected by the current Spanish and European Data Protection Acts.

### 2.3. Samples Processing and Materials

Blood samples were immediately processed by centrifugation through a Ficoll–Hypaque gradient (Pharmacia Corporation, North Peapack, NJ, USA), and peripheral blood lymphocytes (PBMCs) and plasma were isolated and cryopreserved until the moment of analysis. The Raji cell line (ATCC CCL-86) was provided by the existing collection of Instituto de Salud Carlos III (Madrid, Spain). The Vero E6 (African green monkey kidney) cell line (ECACC 85020206) was kindly provided by Dr. Antonio Alcami (CBM Severo Ochoa, Madrid, Spain). Vero E6 and HEK-293T (National Institute for Biological Standards and Control [NIBSC]) cells were cultured in DMEM supplemented with 10% FCS, 2 mM L-glutamine, and 100 units/mL penicillin/streptomycin (Lonza, Basel, Switzerland).

### 2.4. Phenotyping of B Lymphocytes

Subpopulations of B cells (CD3^−^CD19^+^) were analyzed by flow cytometry after staining of surface markers CD10, CD27, CD20, and CD21: immature or transitional cells (CD10^+^ CD27^−^), naïve B cells (CD10^−^CD27^−^CD21^high^), tissue-like memory cells (CD10^−^CD27^−^CD21^low^), resting memory cells (CD10^−^CD27^+^CD21^high^), activated memory cells (CD10^−^CD27^+^CD21^low^), and plasmablasts (CD27^++^CD20^−^CD21^low^) [35]. Antibodies CD3-PE, CD10-BV421, CD19-BV711, CD20-AlexaFluor700, CD21-FITC, and CD27-PercP-Cy5.5 were purchased from BD Biosciences (San Jose, CA, USA). Data acquisition was performed in a BD LSRFortessa X-20 flow cytometer with FACS Diva software (BD Biosciences, San Jose, CA, USA). FlowJo software (Tree Star Inc., Ashland, OR, USA) was used for data analysis.

### 2.5. SARS-CoV-2 Serology

IgG antibodies against the S protein of SARS-CoV-2 were analyzed in plasma samples using the Euroimmun Anti-SARS-CoV-2 ELISA Assay (Euroimmun, Lübeck, Germany). Semi-quantitative results were analyzed by calculating the ratio of extinction of each plasma sample over the calibrator. The results obtained correspond to a semi-quantitative measure calculated as the ratio of the optical density (OD) of each sample over the OD of a calibrator included in the assay. Results were considered positive with IgG titer >1.1; values between 0.8 and 1.1 were considered undetermined, and values <0.8 were considered negative. Borderline data were considered positive.

### 2.6. Pseudovirus Neutralization Assays

One single-cycle, pseudotyped SARS-CoV-2 virus (pNL4-3Δenv_SARS-CoV-2-SΔ19(G614)_Ren) was synthesized by co-transfection of HEK-293T cells with vector pNL4-3Δenv_Ren that expresses HIV-1 genome without env gene and Renilla luciferase gene as reporter [36], together with vector pcDNA3.1-SARS-CoV-2-SΔ19 that expresses G614 SARS-CoV-2 S glycoprotein without the last 19 amino acids (QHU36824.1) [37]. Co-transfection with vector pcDNA-VSV-G, which expressed spike (S) glycoproteins of vesicular stomatitis virus (VSV), was used as control of specificity. The concentration of HIV-1 p24/Gag antigen in cell culture supernatants was quantified 48 h post transfection by Elecsys HIV AG (Roche Diagnostic, Basel, Switzerland).

Plasma neutralization activity was measured by preincubation for 1 h at 37 °C of pNL4-3Δenv_SARS-CoV-2-SΔ19(G614)_Ren pseudovirus (10 ng p24 Gag per well) with fourfold serial dilutions (1/32 to 1/8192) of decomplemented IgG-positive plasma from the recruited patients with haematological disorders and healthy donors as previously described [38]. This mixture was then added to a monolayer of Vero E6 cells and incubated for 48 h. Vero E6 cells were then lysed, and viral infectivity was assessed by measuring Renilla luciferase activity (Renilla Luciferase Assay, Promega, Madison, WI, USA) using a 96-well plate luminometer Centro XS3 LB 960 with MikroWin 2010 software (Berthold Technologies, Baden-Württemberg, Germany). The titers of neutralizing antibodies were represented as 50% inhibitory dose (ID50), which is the highest dilution of plasma that resulted in a 50% reduction of luciferase activity compared to control without serum, using nonlinear regression in GraphPad Prism Software (GraphPad, Inc., San Diego, CA, USA).

### 2.7. Antibody-Dependent Cellular Cytotoxicity Assay

The Raji cell line was used as target to measure ADCC capacity of PBMCs of patients and donors, as described before [39]. Briefly, Raji cells were previously labeled with PKH67 Green Fluorescent Cell Linker (Merck KGaA, Darmstadt, Germany) and then coated with rituximab (50 μg/mL) (Selleckhem, Houston, TX, USA) for 4 h. Labeled Raji cells were then cocultured for 18 h with PBMCs (1:2 ratio) from the recruited patients and healthy donors. Apoptosis of Raji cells was determined by staining with Annexin V conjugated with phycoerythrin (PE) (Immunostep, Salamanca, Spain). Cytotoxic cell populations such as natural killer (NK), NKT-like and TCRγδ+ cells were analyzed in the supernatants using specific conjugated antibodies: CD3-PE, CD56-BV605, CD16-PercP, CD8-APC H7, CD107a-PE-Cy7, and TCRγδ-FITC (BD Biosciences). Data acquisition was performed in a BD LSRFortessa X-20 flow cytometer and FACS Diva software (BD Biosciences). FlowJo software (Tree Star Inc.) was used for data analysis.

### 2.8. Pseudotyped SARS-CoV-2 Infection for Direct Cellular Cytotoxicity Assay

As D614 SARS-CoV-2 viruses were the majority of the earliest variants detected in Spain within clade 19B [40], a mutant clone with D614G change was created by site-directed mutagenesis in pNL4-3Δenv_SARS-CoV-2-SΔ19(G614)_Ren pseudovirus. For analysis of DCC, Vero E6 cells were infected with equal amounts of both one-cycle pseudoviruses D614 and G614 (100 ng p24 Gag/well) and then plated onto 48-well plates. After 48 h of incubation, Vero cells were cocultured for 1 h with PBMCs from patients and healthy donors (ratio 1:10). After detaching Vero monolayer with trypsin–EDTA solution (Sigma Aldrich-Merck, Darmstadt, Germany), caspase-3 activity was measured by luminescence using aCaspase-Glo 3/7 Assay system (Promega). In order to evaluate the effect of cytotoxic cells on the viral replication, Vero E6 cells were lysed, and viral infectivity was assessed by measuring Renilla luciferase activity, as described above. Cytotoxic cell populations such as NK, NKT-like and TCRγδ+ cells were analyzed in the supernatants as described above.

### 2.9. Statistical Analysis

The statistical analyses and graphics were performed using GraphPad Prism 8.0 (GraphPad Software Inc., San Diego, CA, USA). Categorical data were expressed as percentages, and the median was used to describe the central tendency of the numerical data. Group comparisons were performed using the Mann–Whitney U-test, Student’s *t*-test, or Wilcoxon signed-rank test for numerical data, and the chi-square (χ^2^) or Fisher’s exact test for categorical data. A *p*-value (*p*) <0.05 was considered statistically significant in all comparisons.

## 3. Results

### 3.1. Patients’ Characteristics

This was an observational, longitudinal study that included 18 patients diagnosed with NHL (*n* = 16), ITP associated with SLE (*n* = 1), or AIHA associated with DMS (*n* = 1) who received two doses and a booster dose of the authorized mRNA-based COVID-19 vaccine Spikevax (mRNA-1273, Moderna). The main sociodemographic and clinical characteristics of all patients are summarized in Table 1. The median age of the individuals with hematological disease was 61 years (interquartile range (IQR) 53.2–72.8), and most of them (*n* = 12; 66.66%) were female. Individuals with ITP and AIHA received 4 weekly doses of rituximab. Thirteen patients with NHL were treated with four (*n* = 4; 22.22%) and six (*n* = 9; 50%) monthly doses associated with chemotherapy, whereas the remainder (*n* = 5; 27.78%) completed a maintenance scheme with several more doses of rituximab (>8 doses). All individuals with NHL were in complete response at the time of the booster vaccine, and they had not received additional chemotherapy after the last dose of rituximab. The last dose of rituximab was administered at a median of 9.6 months (IQR 6–15) before receiving the second dose of the vaccine Spikevax, and 13.8 months (IQR: 9.4–19) before receiving the booster dose. Patients received a third booster vaccine dose with Spikevax (mRNA-1273, Moderna) within a median of 124.5 days (IQR: 123–128) after the second dose against COVID-19. The first peripheral blood sample was collected the same day all individuals received the booster dose, whereas the second blood sample was collected a median of 28 days (IQR 28.0–33.5) after receiving the booster dose.

In addition, 15 healthy donors who had received two doses and a booster vaccine dose of the authorized mRNA-based COVID-19 vaccine COMIRNATY (BNT162b2, Pfizer-BioNTech) were also recruited. The median age of the healthy donors was 77 years (IQR, 72.0–79.0), and most of them (*n* = 12; 80%) were female. Healthy donors received a third booster vaccine dose within a median of 189.3 days (IQR: 184–189) after the second dose. Similarly to individuals with hematological disease, the first peripheral blood sample was collected the same day they received the booster dose, whereas the second blood sample was collected a median of 29 days (IQR 29.0–42.5) after the booster dose.

### 3.2. Serological Response against COVID-19 Vaccination

**Figure 1:**
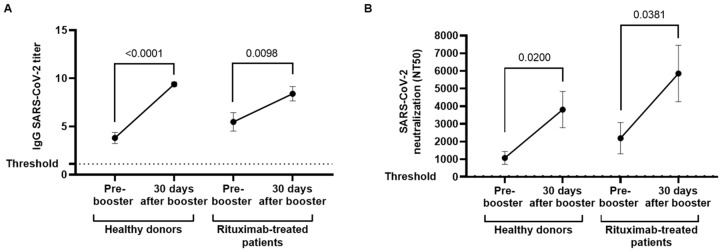
Serological response against COVID-19 vaccine in plasma of rituximab-treated patients and healthy donor before and after receiving the booster dose. (**A**) IgG titers in plasma from individuals with hematological disease who received rituximab before the second dose of vaccine (pre-booster) and the booster dose of SARS-CoV-2 vaccine, in comparison with healthy donors. (**B**) Neutralizing antibody titer at 50% inhibition (NT50) against SARS-CoV-2 of plasma isolated from individuals who received rituximab and healthy donors before and after the booster dose of COVID-19 vaccine. Each dot in the graphs corresponds to mean and the vertical lines correspond to standard error of the mean (SEM). Statistical significance within groups was calculated using the Wilcoxon signed-rank test.

After receiving two doses of mRNA-based COVID-19 vaccine and prior to receiving a booster dose, 13 of 18 (72.22%) individuals of the rituximab-treated cohort developed specific IgGs against SARS-CoV-2 in plasma, in comparison with 14 of 15 (93.33%) healthy donors (Figure 1A). After receiving the booster dose, all (100%) healthy donors and 16 of 18 (88.9%) rituximab-treated patients showed IgGs against SARS-CoV-2 in plasma. Three (16.7%) rituximab-treated patients and one (6.7%) healthy donor, who were seronegative prior the booster dose, seroconverted. One of the individuals that did not seroconvert had received the last dose of rituximab with a concomitant anti-CD19 conjugated antibody (loncastuximab) 40 days prior to the booster, while the other patient received the last treatment of R-CHOP 14 months before.

Titers of specific IgGs against SARS-CoV-2 were increased 2.47-fold (*p* < 0.0001) in healthy donors and 1.53-fold (*p* = 0.0098) in rituximab-treated patients, respectively, 1 month after receiving the booster dose. No significant differences were found between both cohorts before or after receiving the booster vaccine. Similarly, the levels of neutralizing antibodies against SARS-CoV-2 improved after the booster dose in both groups, with a neutralizing capacity 3.78-fold (*p* = 0.0200) higher in healthy donors and 3.03-fold (*p* = 0.0381) in rituximab-treated patients (Figure 1B).

### 3.3. Analysis of B-Cell Subpopulations

**Figure 2:**
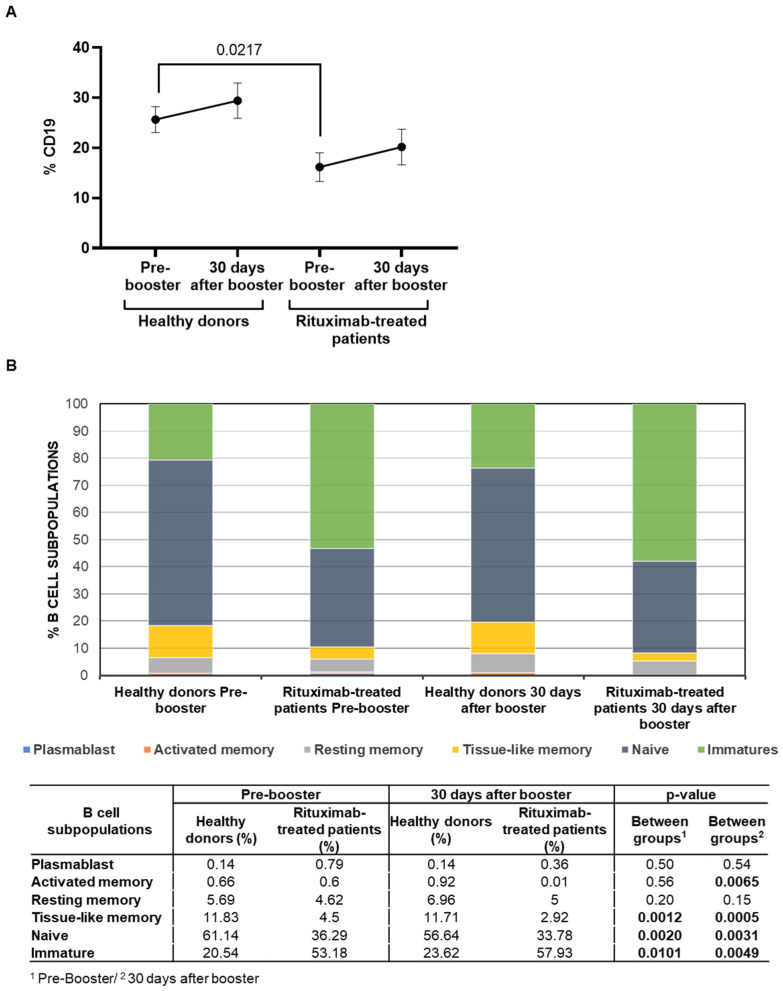
Total levels of B cells and distribution of B-cell subpopulations in PBMCs of rituximab-treated patients and healthy donors before and after receiving the booster dose. (**A**) Total levels of B cells (CD19+) in PBMCs of patients treated with rituximab and healthy donors before and after receiving the booster dose of COVID-19 vaccine. Each dot in the graphs corresponds to the mean ± SEM. Statistical significance was calculated using the Mann–Whitney test. (**B**) Analysis of the distribution of B-cell subpopulations in PBMCs of patients treated with rituximab and healthy donors before and after receiving the booster dose of COVID-19 vaccine. Mean data are represented in bar graphs. Data in the table show the statistical significance between groups that was calculated using Mann-Whitney test.

Total levels of B cells (CD19+) before the booster vaccine were reduced 1.6-fold (*p* = 0.0217) in rituximab-treated patients in comparison with healthy donors (Figure 2A). After receiving the booster, total B-cell levels were not significantly modified in either group. The analysis of B-cell subpopulations showed that rituximab-treated patients presented predominantly an immature phenotype (CD10^+^CD27^−^), with levels that were 2.6-fold (*p* = 0.0101) and 2.5-fold (*p* = 0.0049) higher than those observed in healthy donors before and after receiving the booster dose, respectively. On the other hand, tissue-like memory B-cells (CD10^−^CD27^−^CD21^low^) were reduced 2.6-fold (*p* = 0.012) and fourfold (*p* = 0.0005), in comparison with healthy donors, while the levels of naïve B cells (CD10^−^CD27^−^CD21^high^) were reduced 1.7-fold before (*p* = 0.0020) and after (*p* = 0.0031) receiving the booster dose (Figure 2B).

### 3.4. Antibody-Dependent Cellular Cytotoxic Response of PBMCs from Rituximab-Treated Individuals

**Figure 3:**
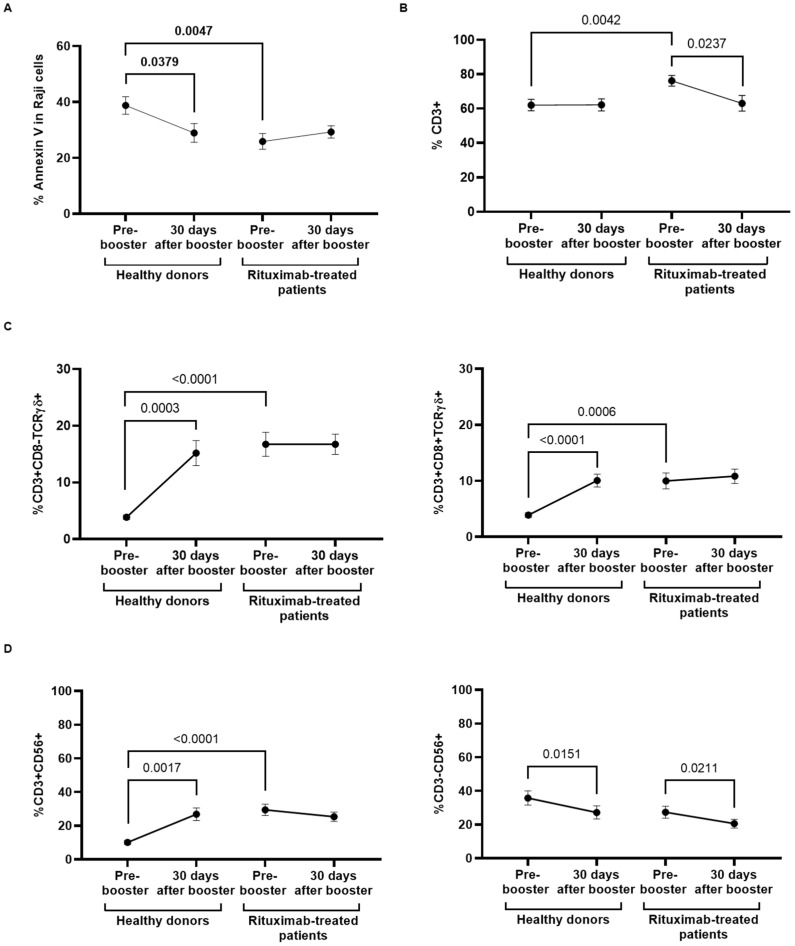
Analysis of ADCC response of PBMCs from rituximab-treated patients and healthy donors before and after receiving the booster dose of COVID-19 vaccine. (**A**) Quantification of the expression of phosphatidylserine in the surface of rituximab-coated Raji cells cocultured with PBMCs isolated from rituximab-treated patients and healthy donors before and after receiving the booster dose, after staining with Annexin V. (**B**) Total levels of CD3+ T cells in individuals of both cohorts that were present in the supernatant of the coculture. (**C**) Levels of CD3+CD8−TCRγδ+ subpopulation (left graph) and CD3+CD8+TCRγδ+ subpopulation (right graph) in PBMCs from individuals of both cohorts. (**D**) Levels of NKT-like cells (CD3+CD56+) (left graph) and NK cells (CD3-CD56+) (right graph) in PBMCs from individuals of both cohorts. Each dot in the graphs corresponds to the mean ± SEM. Statistical significance between groups was calculated using the Mann–Whitney test, and statistical significance within groups was calculated using the Wilcoxon signed-rank test.

ADCC was reduced 1.7-fold (*p* = 0.0047) in rituximab-treated patients 4 months after receiving the second dose of COVID-19 vaccine, in comparison with healthy donors, and the booster dose did not improve this response in either group of participants (Figure 3A). The analysis of the cytotoxic cell populations that could be responsible for this ADCC response showed that total T-cell levels (CD3+) were increased 1.2-fold (*p* = 0.042) in rituximab-treated individuals compared to healthy donors previous to the booster dose, but these levels were reduced (*p* = 0.0237) after the booster dose (Figure 3B). We did not find changes in the levels of total CD8+ T lymphocytes after receiving the booster dose in any group of participants (data not shown [41]). However, the levels of highly cytotoxic populations of CD3+CD8−TCRγδ+ and CD3+CD8+TCRγδ+ cells were increased 3.8-fold (*p* < 0.0001) and 2.2-fold (*p* < 0.0006), respectively, in rituximab-treated patients in comparison with healthy donors, prior to receiving the booster dose (Figure 3C). The levels of these cells remained unchanged in rituximab-treated individuals after receiving the booster dose, whereas they were significantly increased in healthy donors 3.94-fold (*p* = 0.0003) and 2.59-fold (*p* < 0.0001), respectively. The same pattern was observed in the level of NKT-like cells (CD3+CD56+) that was increased 2.7-fold (*p* < 0.0001) in rituximab-treated patients before receiving the booster dose, in comparison with healthy donors (Figure 3D, left graph). The level of these cells remained unchanged in rituximab-treated individuals after the booster dose, but it was increased 1.4-fold (*p* = 0.0017) in healthy donors. The levels of NK cells (CD3−CD56+) were similar in both cohorts, and they showed a significant decrease after receiving the booster dose (Figure 3D, right graph). The activation of these cytotoxic cells was evaluated through the expression of the degranulation marker CD107a, but it remained stable in both cohorts before and after the booster dose (data not shown [41]).

### 3.5. Specific Direct Cellular Cytotoxicity of PBMCs and Viral Neutralization

**Figure 4:**
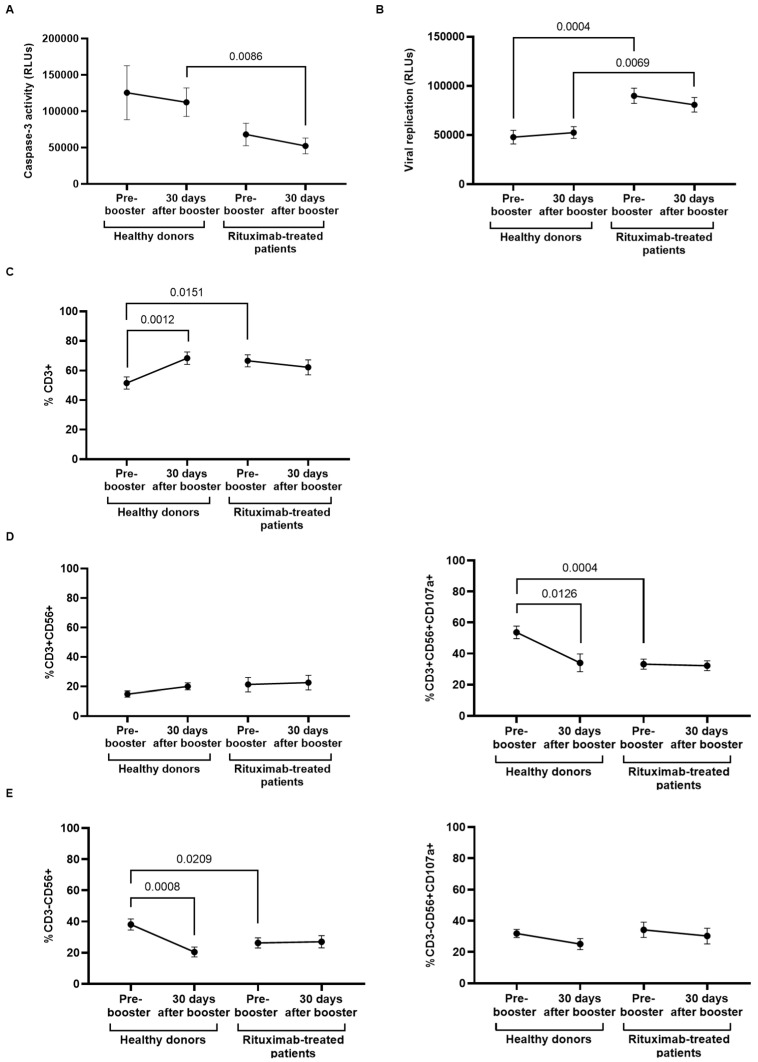
Analysis of DCC response of PBMCs from rituximab-treated patients and healthy donors before and after receiving the booster dose of COVID-19 vaccine. (**A**) DCC was assessed by measuring the activity of caspase-3 in pseudotyped-SARS-CoV-2-infected Vero E6 cells cocultured with PBMCs from rituximab-treated patients in comparison with healthy donors before and after the booster dose. (**B**) The capacity of PBMCs from both cohorts to eliminate pseudotyped SARS-CoV-2-infected Vero E6 cells was determined by quantifying the production of renilla (relative light units, RLUs) after coculture for 1 h. (**C**) Total levels of CD3+ T cells in individuals of both cohorts that were present in the supernatant of the coculture. (**D**) Total levels of NKT-like cells (CD3+CD56+) (left graph) and NKT-like cells expressing the degranulation marker CD107a (CD3+CD56+CD107a+) (right graph) in PBMCs from individuals of both cohorts. (**E**) Total levels of NK cells (CD3−CD56+) (left graph) and NKT-like cells expressing the degranulation marker CD107a (CD3−CD56+CD107a+) (right graph) in PBMCs from individuals of both cohorts. Each dot in the graphs corresponds to the mean ± SEM. Statistical significance between groups was calculated using the Mann–Whitney test, and statistical significance within groups was calculated using the Wilcoxon signed-rank test.

The specific DCC response against Vero E6 cells infected with pseudotyped SARS-CoV-2 of PBMCs isolated from rituximab-treated patients and healthy donors did not significantly change one month after receiving the booster dose, but this activity was reduced 2.0-fold (*p* = 0.0086) in PBMCs of rituximab-treated patients in comparison with healthy donors (Figure 4A). This diminished cytotoxic activity caused a decreased capacity of PBMCs from rituximab-treated individuals to neutralize the viral replication after coculture with the infected cells, which was 2.0-fold (*p* = 0.0004) and 1.6-fold (*p* = 0.0069) reduced before and after receiving the booster dose, respectively, in comparison with healthy donors (Figure 4B). The analysis of the cell populations that could be responsible for the DCC response showed that total T-cell count was 1.2-fold (*p* = 0.0151) higher in PBMCs from rituximab-treated patients; however, it remained steady after the booster dose, whereas it was increased 1.3-fold (*p* = 0.0012) in healthy donors (Figure 4C). Similarly, the booster dose did not increase the levels of NKT-like (CD3+CD56+) cells (Figure 4D, left graph) or NK (CD3−CD56+) cells (Figure 4E, left graph) in rituximab-treated individuals and healthy donors. However, the expression of CD107a in NKT-like cells of rituximab-treated patients was diminished 1.5-fold (*p* = 0.0004) before receiving the booster dose in comparison with healthy donors, and it did not significantly change after the booster dose (Figure 4D, right graph). The booster did not improve the activity of NKT-like cells in healthy donors either. In addition, the level of NK cells was reduced 1.3-fold (*p* = 0.0209) in rituximab-treated individuals, in comparison with healthy donors, before receiving the booster dose, and it did not change after the booster dose. NK cell degranulation capacity was not modified after the booster dose (Figure 4D, left graph). No significant differences were found in CD8+ T-cell count, in the levels of TCRγδ+ cells, or in the expression of CD107a in these cells between both cohorts, before or after receiving the booster dose (data not shown [41]).

### 3.6. Breakthrough Infections

Six of 18 (33.33%) rituximab-treated patients had a breakthrough infection with SARS-CoV-2 within a median time of 92 days (IQR: 70–134) since receiving the booster vaccine dose. Infection was determined by positive antigen COVID-19 test or specific RT-qPCR. Five (83.4%) infected patients suffered mild infection that did not require hospitalization. One (16.6%) patient required admission to the hospital and low-oxygen-flow support before complete recovery without further complications. This patient had received the last dose of rituximab more than 1 year before receiving the booster and presented proper neutralizing antibodies titers after the second dose against SARS-COV-2 that were stable after the booster vaccine. There were no infections reported in the healthy donor group.

## 4. Discussion

Individuals with hematological diseases on treatment with immunosuppressive or anticancer agents are more susceptible to developing severe forms of COVID-19, due to the characteristics of the disease or the treatment they received [42]. Therefore, the vaccination of this population has been a priority since the authorization of the first COVID-19 vaccines [12]. Most studies about the efficacy of COVID-19 vaccines have been focused on the analysis of the humoral response, although some studies have also evaluated the cellular response at 6 months following a completed primary series of vaccination. Although elevated T-cell responses have been observed in most individuals at 6 months, the results have varied from a 42% to a 90% increase [43], and there is still controversy about the correlation among these increased levels of T cells, the development of memory B cells, and the presence of neutralizing antibodies. Anti-CD20 antibodies such as rituximab are known to efficiently cause a peripheral B-cell aplasia that begins to recover 6 to 9 months after the last dose of the antibody [3,44]. This aplasia, along with other treatments that may be associated with the anti-CD20 antibodies in the patients, such as chemotherapy, or the disease itself, confers high immunosuppression. Consequently, there is major concern about the efficacy of COVID-19 vaccines in individuals treated with B-cell-depleting immunotherapy, as the experience with other vaccines has shown an impaired humoral response [1,2,3]. Recent studies confirmed that seroconversion in individuals on treatment with anti-CD20 after receiving two doses against COVID-19 is suboptimal compared with healthy donors (<10%) [45,46]. This percentage significantly increases after ceasing the treatment, from 17.5% in patients that ended anti-CD20 less than 6 months earlier [47] to higher results of 66–80% [28,45,47,48] 6–9 months since the last dose, which also concurs with the time of peripheral B-cell aplasia recovery. Not only are seroconversion and titers decreased in these patients, but so is the neutralizing capacity of these antibodies, which was impaired even 12 months after having received rituximab [49]. Consequently, rituximab-treated individuals present a high risk for mortality, severe disease, and prolonged in-hospital stay after SARS-CoV-2 infection [50], and the full vaccination schedule presents highly variable efficacy that is mostly influenced by the time that has passed since the last dose of rituximab.

In immunocompetent patients and solid organ transplant patients, the booster vaccine dose against COVID-19 has demonstrated clinical and serological efficacy [51,52], but data regarding the improvement of this humoral immune response in patients treated with anti-CD20 are still scarce. Preliminary results in small series of patients suggest that individuals with active anti-CD20 treatment do not improve their humoral response after the booster vaccine but are likely to seroconvert if they had discontinued immunotherapy for more than 1 year [53,54]. However, these results focused only on the humoral response; therefore, there is limited information about the cellular immune response or about the integration of both humoral and cellular immune responses in this population, which is essential to control SARS-CoV-2 infection and avoid severe disease [31,32,55]. This is particularly interesting in individuals treated with rituximab, as it seems to be a significant dissociation between humoral and cellular immune responses, presenting T-cell responses (71–85%) that do not rely on seropositivity [28,45,47,54,56]. Nonetheless, these results derive from the quantification of the release of proinflammatory cytokines such as IFNγ, TNFα, or IL-2, after stimulation with SARS-CoV-2 peptides, but they do not provide any information about the capacity to generate an effective cytotoxic response against SARS-CoV-2-infected cells that may contribute to clearing the infection.

In this study, we presented a cohort of patients that had been recently treated with rituximab due to their hematological disease. They received the full vaccination schedule against COVID-19 with Spikevax (mRNA-1273, Moderna) within a median of 9.6 months after receiving the last dose of rituximab. A group of 15 healthy donors were used as controls, and they had been fully vaccinated with COMIRNATY (BNT162b2, Pfizer-BioNTech). The efficacy for these vaccines has been analyzed in several clinical trials and real-world studies [17,27] that have determined similar results for both vaccines, being estimated at 92–98% efficacy against SARS-CoV-2 infection after receiving two doses [57,58]. There have been major concerns about the efficacy against the new variants of SARS-CoV-2, since these variants have multiple mutations, including those in the S protein of the virus that may change the effectiveness of the different vaccines [59]. Even though protection against the Delta variant infection seems to decrease to 53.5–88% with two doses of COMIRNATY and 76–78% with two doses of Spikevax, prevention of severe infection and death remains adequate with 89–93.1% and 90–95%, respectively [16,60]. Results for the Omicron variant showed only 33% of protection against infection, with an increased rate of breakthrough infections that were mostly mild [61]. According to this information, the results obtained from both cohorts of patients and controls would be comparable.

We analyzed that both humoral and cellular early immune responses produced approximately 4 months after receiving the second dose of vaccine and 1 month after receiving the booster dose, which was administered a median of 13.8 months after the last dose of rituximab. The information obtained could be essential to get a better understanding about the development of the immune response after COVID-19 vaccination in these individuals and the real benefit of the booster vaccine. In accordance with previously published reports, the individuals from our cohort showed levels of seroconversion and neutralizing capacity that were comparable to healthy donors 4 months after receiving the second dose of vaccine, despite the presence of a certain level of aplasia with a significantly higher count of immature B cells. Interestingly, these levels were further increased 1 month after receiving the booster dose, which indicated the development of a functional memory B-cell response 13 months after the last dose of rituximab. Moreover, rituximab-treated individuals who were seronegative after receiving the second vaccine dose developed adequate IgG titers with neutralizing capacity after receiving the booster. Therefore, extending the period without immunotherapy treatment enabled an optimal seroconversion as those patients who had received the last dose of anti-CD20 more than 9 months prior to the booster dose developed a humoral response that was comparable to healthy donors.

The role of IgGs in protecting against SARS-CoV-2 infection relies not only on their neutralizing capacity to protect cells from infection, but also on their ability to activate the complement system and ADCC response exerted by NK and CD8+ T cells to eliminate the infected cells in case of breakthrough infections. We observed that the booster dose significantly increased the levels of highly cytotoxic cells such as CD3+CD8±TCRγδ+ and NKT-like cells in PBMCs from healthy donors, although it did not correlate with an increase in the ADCC response that was previously developed after the second vaccine dose. In rituximab-treated individuals, ADCC response was significantly reduced in comparison with healthy donors, and it did not improve after the booster dose. This could be due to the cell counts or to the fact the level of activation of the essential cytotoxic population that may mediate ADCC in these patients was not modified by the booster dose, which could be related to the residual aplasia and/or to the basal high levels of some of these subpopulations, such as TCRγδ cells, which is usually observed in oncohematological patients as a consequence of the disease [62,63,64]. Similar results were obtained when DCC activity against cells infected with pseudotyped SARS-CoV-2 was analyzed. PBMCs from rituximab-treated individuals showed a decrease in DCC activity in comparison with healthy donors 4 months after having received the second dose, and this activity was not improved after receiving the booster dose. As a result, the overall capacity of PBMCs from rituximab-treated individuals was unable to clear the infected cells even after the booster dose. Interestingly, although the booster dose induced an overall increase in the level of total CD3+ T cells in healthy donors, we did not observe an improvement in any of the analyzed cytotoxic cell populations. This lack of enhancement in the levels and activity of cytotoxic cells was also observed in rituximab-treated individuals before and after receiving the booster dose. This translates into a suboptimal cytotoxic capacity and, consequently, an impaired DCC in rituximab-treated individuals, which may be related to other treatments that are usually associated with rituximab such as chemotherapy in NHL patients or other immunosuppressives drugs in IT and AIHA patients. In fact, 14 (78%) individuals had received a concomitant treatment within the last 18 months prior to the booster, including a high dose of corticosteroids in 12 of them, which holds a strong immunosuppressive effect even in monotherapy [65]. However, corticosteroids are often combined with other chemotherapy regimens in NHL, such as CHOP or bendamustine, which depletes hematopoietic precursor lineages, especially B- and T-cell lymphoid cells [66]. This induces more profound and long-term immunosuppression in these patients; for example, alkylate drugs, such as bendamustine, generate a prolonged CD4 lymphopenia which decreases up to 6 months after receiving the last dose and does not recover until 7–9 months after the end of treatment [67,68,69,70]. Some studies stated that this lymphopenia is more profound when bendamustine is combined with rituximab [71]. Not only may systemic treatments contribute to a suboptimal immune response, but local radiotherapy may also impair proliferation of the hematological lineages in the bone marrow, causing abnormal T-cell development in the thymus or even transient thymus atrophy [66], lymphopenia, and T dysregulation [72,73,74].

Breakthrough infections within 2–6 months following two doses of COMIRNATY or Spikevax are relatively low, and several studies have calculated that infections are less frequent in vaccinated healthy individuals (0.2–6.3% across studies) than in unvaccinated individuals (2.2–7.5% across studies) [75,76,77,78]. Taking into account these data, the rate of breakthrough infections detected in our cohort (six of 18; 33.33%), acquired a median time of approximately 3 months after receiving the booster dose, was considerably higher. Although most of these individuals (five of six; 83.33%) had mild infection and only one (one of six; 16.67%) required hospitalization, these data showed that the immune protection exerted by the booster dose in rituximab-treated individuals may not last as long as in untreated, healthy individuals.

## 5. Conclusions

In conclusion, we determined that the booster dose of Spikevax improved the humoral immune response in rituximab-treated patients, similarly to the booster dose of COMIRNATY in healthy donors. However, it has been observed that the levels of IgG against SARS-CoV-2 progressively decreased within 6 months after receiving the second vaccine dose in immunocompetent individuals [22]. Therefore, it remains to be seen if a booster dose may elicit a perdurable humoral response, not only in immunocompetent but also in immunocompromised individuals. Interestingly, the booster dose did not significantly modify the cellular immune responses elicited by the second dose in healthy individuals. Moreover, these cellular responses were reduced in rituximab-treated individuals and did not improve after receiving the booster dose. However, although breakthrough infections were higher in our cohort of rituximab-treated patients than in healthy donors, most of them developed a mild form of COVID-19, likely due to the high levels of neutralizing antibodies elicited by Spikevax booster dose. Therefore, although the cellular responses appeared to be developed suboptimally in rituximab-treated individuals after three doses of Spikevax, the excellent humoral response developed despite the low levels of B cells and the altered distribution of B-cell subpopulations was able to prevent infection in most individuals. Due to the potent residual effect of rituximab on both humoral and cellular immunity, it is essential to further investigate the effect of the booster dose in individuals who are on active treatment with rituximab or who have discontinued it recently, as this benefit over the humoral response observed in the individuals from our cohort may be reduced in those patients who received rituximab within 6 months before the booster dose.

## Figures and Tables

**Table 1:** Baseline sociodemographic and clinical characteristics of the individuals with hematological disease who were recruited for this study.

	Patients (*n* = 18)	Healthy Controls (*n* = 15)
Age, median (IQR)	61 (53.2–72.8)	77 (72.0–79.0)
Sex: Female, *n* (%)	12 (66.66)	12 (80%)
Diagnosis		
- NHL, *n* (%) - AIHA, *n* (%) - IT/SLE, *n* (%)	16 (88.89) 1 (5.56) 1 (5.56)	- - -
Number of doses of rituximab, *n* (%)		
- 4 doses - 6 doses - >8 doses	4 (22.22) 9 (50) 5 (27.78)	- - -
Concomitant chemotherapy within 18 months prior to booster, *n* (%)		
- None - Prednisone - CHOP - Bendamustine - Loncastuximab - Radiotherapy - Methotrexate	4 (22.22) 2 (11.11) 10 (55.56) 2 (11.11) 1 (5.56) 2 (11.11) 1 (5.56)	- - - - - - -
Months from last treatment with rituximab to second vaccine dose, median (IQR)	9.6 (6–15)	-
Months from last treatment with rituximab to third vaccine dose, median (IQR)	13.8 (9.4–19)	-
Days from second dose to first sample, median (IQR)	124.5 (122.75–126)	189.3 (184–189)
Days from booster dose to second sample, median (IQR)	28 (28–33.5)	29 (29.0–42.5)

AIHA, autoimmune hemolytic anemia; CHOP, cyclophosphamide, doxorrubicine, vincristine, prednisone; IQR, interquartile range; IT, immune thrombocytopenia; NHL, non-Hodgkin’s lymphoma; SLE, systemic lupus erythematosus.

## Data Availability

The original contributions presented in the study are included in the article. Further inquiries can be directed to the corresponding authors.
